# The effectiveness of expanded carrier screening based on next-generation sequencing for severe monogenic genetic diseases

**DOI:** 10.1186/s40246-024-00577-w

**Published:** 2024-01-31

**Authors:** Xue Zhang, Qian Chen, Junnan Li, Xin Luo, Jianyun Luo, Jian Li, Ziye Zeng, Yan Wu, Hua Zhang, Yanling Dong

**Affiliations:** Department of Obstetrics, The First Affiliated Hospital of Chongqing Medical University, No. 1, Youyi Road, Yuanjiagang, Yuzhong District, Chongqing, 400016 People’s Republic of China

**Keywords:** Expanded screening carrier, Next-generation sequencing, Monogenic genetic diseases

## Abstract

Expanded carrier screening (ECS) based on next-generation sequencing has been the subject of few studies to estimate the effectiveness of ECS in the Chinese population. A total of 3737 individuals from Southwest China or the general Chinese population, including 1048 pairs and 1641 individuals, were analysed by ECS for 155 monogenetic diseases. An ECS panel was used to detect 147 genes and 10,449 variants in 145 autosomal recessive and 10 X-linked recessive disorders. A total of 43.27% (1617/3737) were found to be carriers of at least one of the 155 monogenetic diseases. The average number of carriers of these recessive mutations was 0.54 and ranged from 0 to 4. Of the 1048 couples, 74.81% (*n* = 784) were found to have at least one partner carrying more than one disease. In addition, 5.34% of the couples at risk (*n* = 56) were heterozygous for the same autosomal recessive disease, and 0.37% of the women (9/2440) were carriers of X-linked diseases. Our study demonstrated the clinical significance of ECS in Chinese populations and the need for a programme of familial screening for the prevention of severe recessive monogenetic diseases.

**Supplementary Information:**

The online version contains supplementary material available at 10.1186/s40246-024-00577-w.

## Introduction

Mendelian diseases may be individually rare but they are leading causes of neonatal morbidity and mortality, and in recent studies, 36.7–57.0% of critically ill infants were molecularly diagnosed with Mendelian diseases through rapid clinical exome sequencing or whole-genome sequencing [1]. Both parents or the mothers of affected individuals with autosomal recessive (AR) or X-linked recessive diseases, respectively, are carrier(s). Carrier screening was first conducted for Tay-Sachs disease in 1971 [2]. After more than 40 years of clinical experiences, more than 448 recessive diseases were selected for the screening using next-generation sequencing (NGS) [3]. Expanded carrier screening (ECS) based on NGS goes beyond traditional carrier screening methods which typically focuses on common mutations associated with more well-known disorders. NGS technologies allow for rapid and cost-effective analysis of large amounts of DNA sequence data making this type of screening feasible at a population level [4].

ECS benefits prenatal screening and informed prenatal testing options such as preimplantation genetic diagnosis, prenatal invasive testing, and other reproductive options. Couples with carrier of most recessive genetic disease are typically asymptomatic, and the only way to identify them is by the carrier screening. And ECS provides an effective way to identify couples at risk by recessive genetic diseases, including autosomal and X-linked diseases. If examine during pregnancy period, the male partner should be test at the same time. It will be less stressful on patients with positive results if they screened on the preconception than prenatal screening [5, 6]. For the AR disease, the offspring of a couple has 25% chance of being affected if both parents are carriers of the diseases. For the X-linked inherited condition, the male offspring of female has the 50% chance of being affected if the mother is a carrier. Since finding carriers can prevent severe diseases and birth defects in babies by simple lifestyle changes during pregnancy, ECS based on NGS makes sense both medically and economically.

Guidelines by the American College of Obstetricians and Gynecologists (ACOG) and the American College of Medical Genetics and Genomics (ACMG) suggest making full consideration of population characteristics when application of ECS [7, 8]. According to the guidelines set forth by the ACOG and the American College of Medical Genetics and Genomics (ACMG), population characteristics such as ethnicity, geographic origin, and socioeconomic status should be considered when considering the use of ECS. These factors can influence the frequency of specific genetic conditions in certain populations and may impact the likelihood of finding carriers within those groups.

The birth defect rate in China is estimated to be 5.6%, of which 22% are caused by monogenic genetic diseases [9]. However, there have been few studies on the estimation of the effectiveness of ECS in this population. In the current study, a single-centre retrospective analysis was conducted on 3737 individuals (including 1048 couples) in the First Affiliated Hospital of Chongqing Medical University of China. A total of 155 monogenic recessive diseases, including 147 genes, were carefully selected to be analysed by an expanded carrier NGS screening panel. We analysed the carrier frequencies of these diseases and found the top 10 most common gene recessive diseases. We also found 56 couples and nine female X-linked disease carriers for barrier offspring with a recessive disease. Our findings suggest a need to promote ECS in clinical settings in the region.

## Methods

### Study subjects

A retrospective analysis was conducted from April 2022 to April 2023 at the First Affiliated Hospital of Chongqing Medical University in China. Regarding the inclusion criteria for this screening, this screening is a general population screening programme with no special restrictive criteria and is suitable for all pre-pregnancy and early pregnancy couples who intend to have children. In this study, phenotypically normal couples were screened at the same time, with the woman being examined first on a voluntary basis. Basic clinical information such as age, pregnancy status, gestational age, and family history were collected. Written informed consent was obtained from the pregnant women for publication of clinical information and genetic results. The study was approved by the Ethics Committee of the First Affiliated Hospital of Chongqing Medical University (approval number K2023-248).

### Targeted next-generation sequencing (panel) and data analysis

Genomic DNA was extracted from peripheral blood using the DNA Extraction Reagent Kit (The Beijing Genomics Institute, China). The Library Construction and Hybridisation Capture Kit (BGI, China) was used for capture, enrichment, and elution. The DNA was then sequenced on a commercially available targeted next-generation sequencing (panel) called MGISEQ-2000 (BGI, China). The detected loci form the BGI Commercially available kit was selected with reference to the requirements of the international ACMG, ACOG, and other professional societies, and in accordance with the following principles: (1) The disease has a high incidence rate and a high carrier rate in the Chinese population; (2) the disease’s causative gene is clearly identified and clearly associated with its phenotype; (3) the disease has a serious impact on the population, resulting in death, teratogenicity, disability, and stupidity, and requires timely surgical or medical interventions; (4) the disease mostly develops during the foetal period and neonatal and childhood; (5) the disease is preventable and blockable, and the clinical outcome can be improved through prenatal diagnosis or delivery management; and (6) the disease is preventable and interruptible, and clinical outcomes can be improved through prenatal diagnosis or delivery management. All the test loci in the database have been strictly interpreted and audited by professional personnel.

According to the published article, targeted region capture, enrichment, elution, and data analysis were performed as follows [10]. The target gene of this panel was arrived by comparing the whole-genome data of Asia, comprehensively evaluating the carrier frequency and the inclusion and exclusion criteria of the international guidelines [8]. Firstly, the genome-wide data of Chinese people were used to comprehensively evaluate the incidence rate of carriage; top 300 diseases were screened for carriage; and experts in the field were invited to evaluate and rank the diseases, and then, 155 diseases were finally screened according to the inclusion and exclusion criteria of the international guidelines [8]. Disease and genes are shown in Additional file 1: Table 1. The capture of target regions, enrichment, and analysis were performed according to the previously published protocol [11], and the performance of the screening assay was evaluated according to the published study [12].

After the data were off-loaded, the information was analysed. Firstly, the sequencing quality of the offline raw data (raw reads) was evaluated to remove low quality and reads contaminated by connectors, and then, the sequence comparison with Hg19 was performed by BWA software (Burrows Wheeler Aligner), and at the same time, the sequence capture effect was evaluated, and the statistics of the depth and coverage, and the single-base depth distribution maps were plotted. SNV (single-nucleotide variant) and Indel (insertion and deletion) queries were performed using samtools 1.13 and gatk 4.1.9.0 software, respectively, to generate the results of base polymorphisms in the target region, followed by database comparison and annotation of the identified suspicious mutations, screening. The sensitivity, specificity, accuracy, positive predictive value, and negative predictive value of SNVs on the targeted regions reached 99.9%, when sequencing depth was greater than 100-fold. The coverage of targeted regions was 99.83%. The percentage of average depth in subjects and exon (> 20-fold) was 99.47% and 99.96%, respectively. In general, on the basis of the validation results, a mean sequencing depth of ≥ 100-fold was aimed for in the study of 3737 patients. The positive mutations in this ECS panel were the known pathogenic and likely pathogenic mutations. The screening loci were interpreted with reference to the American College of Medical Genetics and Genomics (ACMG) guidelines.

### Validation of results

Point mutations were validated by Sanger, for CNVs in alpha-thalassemia by gap-PCR, and other genes of CNVs were validated by qPCR or MLPA. All positive results were confirmed. For example, for SMA, our screen currently detects exon 7 of SMN1; for DMD, it detects not only point mutations and small insertional deletions in the DMD gene, but also CNV, and the CNV results in the report were verified by qPCR or MLPA. For thalassaemia genes, the intronic and promoter regions were also detected, and large fragment deletions in the alpha-thalassaemia HBA/HBB genes—SEA,—alpha 3.7,—alpha 4.2, Chinese, SEA-HPFH, FIL,—THAI, and Taiwanese were also included.

### Statistical analysis

Data are presented as the mean ± standard deviation using SPSS version 17.0 (SPPS, Inc.). Statistical significance between > 2 groups was determined using Botteron’s test after one-way ANOVA, and a *t*-test was used to compare two groups. *P* < 0.05 was considered to indicate a statistically significant difference.

## Results

### Population demographics

The screening population included 3737 individuals, including 1048 couples and 1641 individuals. There are 2440 female and 1297 male subjects. The subjects were from the obstetrics department (*n* = 3378, 90.39%) and the assisted reproduction department (*n* = 359, 9.61%) for preconception screening or pre-pregnancy examination (as shown in Table 1). A total of 3378 subjects were from the obstetrics unit (90.39%) and 359 from the assisted reproduction unit (9.61%). These two departments received similar numbers of couples and individuals. The age of the patients ranged from 19 to 56 years, with an average age of 31.57 years. A total of 1241 patients were pregnant. The gestational age and percentage of those who were pregnant are shown in Table 1. In addition, there were only two consanguineous couples and one individual. In these two couples, one partner was a carrier of one variant, and the other was negative. The individual's screening result was negative.

**Table 1:** Demographics of subjects

Demographics	*n*	% of total
Total	3737	
Obstetrics	3378	90.39
Couples	948	56.13^a^
Singulars	1482	43.87^a^
Assisted reproduction	359	9.61
Couples	101	52,65^b^
Singulars	157	47.35^b^
Patient’s age (years)		
≤ 24	98	2.62
25–29	1411	37.76
30–34	1432	38.32
35–39	594	15.90
≥ 40	202	5.41
Pregnancy status of female (weeks)		
Not pregnancy	1199	49.14^c^
Pregnancy	1241	50.86^c^
≤ 13	525	42.30^d^
14–28	481	38.76^d^
≥ 29	235	18.94^d^

^a^Percentage of 3378 subjects from the Obstetrics Department

^b^Percentage of 359 subjects from the Assisted Reproduction Department

^c^Percentage of 2440 female subjects

^d^Percentage of 1241 pregnancy female subjects

### Distribution of frequent pathogenic gene and carrier frequencies

A total of 3737 individuals, including 1048 couples and 1641 individuals, underwent carrier screening for 155 recessive diseases (in 147 genes). About 43.27% (*n* = 1617) of the population were carriers of at least one of the 155 diseases. Of 1048 couples, 264 couples (25.19%) were negative on screening, and 56 couples (5.34%) were carriers of the same AR gene (Table 2).

**Table 2:** The at risk of couples and female

Disease	*n*	Gene*	Variants	Frequency (%)
AR	56			5.34^a^
DFNB1A	27	*GJB2*	c.109G > A, c.109G > A	2.55^a^
	10		c.235delC, c.109G > A	
	2		c.235delC, c.235delC	
	1		c.109G > A, c.139G > T	
	1		c.583A > G, c.109G > A	
	1		c.508_511dupAACG, c.235delC	
α-thalassemia	1	*HBA1/2*	-alpha 3.7, -alpha 3.7	0.38^a^
	2		-SEA, -alpha 3.7	
	1		-alpha 3.7, -SEA	
PCD	1	*SLC22A5*	c.1195C > T, c.760C > T	0.19^a^
	1		c.1400C > G, c.1195C > T	
Phenylketonuria	1	*PAH*	c.472C > T, c.1174 T > A	0.19^a^
	1		c.721C > T, c.721C > T	
OCA	1	*OCA2*	c.2359G > A, c.1426A > G	0.09^a^
SMA	1	*SMN1*	EX7 DEL, EX7 DEL	0.09^a^
Joubert syndrome	1	*CC2D2A*	c.2848C > T, c.4583G > A	0.09^a^
HLH	1	*PRF1*	c.65delC, c.1228C > T	0.09^a^
LGMD	1	*DYSF*	c.895G > A, c.2875C > T	0.09^a^
BH4D	1	*PTS*	c.155A > G, c.186 + 1G > A	0.09^a^
XL	9			0.37^b^
DMD	1	*DMD*	EX45_51DEL	0.25^b^
	1		c.8547 + 2 T > G	
	1		EX45_51DEL	
	1		EX1_4DUP	
	1		c.2776C > T	
	1		c.1615C > T	
MPS II	1	*IDS*	c.200 T > G	0.08^b^
	1		c.459delG	
Haemophilia B	1	*F9*	c.316G > A	0.04^b^

^*^DFNB1A, Autosomal Recessive Deafness 1A; SMA, Spinal Muscular Atrophy; OCA, Oculocutaneous Albinism; PCD, Primary Carnitine Deficiency; HLH, Haemophagocytic Lymphohistiocytosis, Familial, 2; LGMD, Limb-Girdle Muscular Dystrophy Type 2B; BH4D, Tetrahydrobiopterin Deficiency; DMD, Duchenne Muscular Dystrophy; and MPS II, Mucopolysaccharidosis Type II

^a^Percentage of 1058 couples screened

^b^Carrier frequency in 2440 female subjects

Of the 3737 individuals screened, 43.27% (*n* = 1617) were found to carry at least one disease. Of the 1617 carriers, 78.79% (*n* = 1274) were found to be carriers of one disease, and 21.21% (*n* = 343) were found to be carriers of two or more diseases. About 74.81% (*n* = 784) of the 1048 couples were positive for at least one disease. Otherwise, 5.34% (*n* = 56) of high-risk couples were heterozygous for the same AR disease.

A total of 128 (82.58%) recessive diseases were identified in 3737 subjects. The carrier frequencies of the top 10 conditions are summarised in Table 3. The total carrier frequency of the top 10 conditions is 32.22% (1204/3737). The most common condition is autosomal recessive deafness 1A (DFNB1A) with a rate of 12.95% (*n* = 484), which is exceptionally high compared to other conditions detected in this cohort. It was almost several times higher than α-thalassemia (*n* = 186, 4.98%) and Wilson disease (*n* = 108, 2.89%). The carrier frequencies of other diseases were autosomal recessive deafness 4 (DFNB4) (*n* = 78, 2.09%), spinal muscular atrophy (SMA) (*n* = 77, 2.06%), β-thalassemia (*n* = 68, 1. 82%), primary carnitine deficiency (PCD) (*n* = 66, 1.77%), phenylketonuria (*n* = 64, 1.71%), Krabbe disease (*n* = 37, 0.99%), and MUT-related methylmalonic acidaemia (*n* = 36, 0.96%).

**Table 3:** Carrier frequencies of top 10 recessive diseases

Number	Disease	Gene*	Cases	Carrier frequency^a^ (%)
1	DFNB1A	*GJB2*	484	12.95
2	α-thalassemia	*HBA1/HBA2*	186	4.98
3	Wilson disease	*ATB7B*	108	2.89
4	DFNB4	*SLC26A4*	78	2.09
5	SMA	*SMN1*	77	2.06
6	β-thalassemia	*HBB*	68	1.82
7	PCD	*SLC22A5*	66	1.77
8	Phenylketonuria	*PAH*	64	1.71
9	Krabbe disease	*GALC*	37	0.99
10	MMA	*MMUT*	36	0.96
Total			1204	32.22

^*^DFNB1A, Autosomal Recessive Deafness 1A; DFNB4, Autosomal Recessive Deafness 4; SMA, Spinal Muscular Atrophy; PCD, Primary Carnitine Deficiency; and MMA, MUT-Related Methylmalonic Acidaemia

^a^Percentage of 3737 individuals screened

### The at-risk couples/female subjects

If the couple are both carriers of the same autosomal recessive disease, or the female is a carrier of an X-linked recessive disease, they are identified as a risk couple. They are more likely to have an affected pregnancy. As shown in Table 2, 56 carrier couples (5.34%) were heterozygous for a disease. DFNB1A was the most common disease in risk couples (*n* = 42). Four couples with α-thalassemia and two couples with PCD and phenylketonuria were studied separately. One pair of oculocutaneous albinism (OCA), SMA, Joubert syndrome, haemophagocytic lymphohistiocytosis, familial 2 (HLH), limb-girdle muscular dystrophy type 2B (LGMD), and tetrahydrobiopterin deficiency (BH4D) were also studied separately. In addition, 9 females (0.37%) were carriers of X-linked recessive diseases, including one for haemophilia B, two for mucopolysaccharidosis II, and six for Duchenne muscular dystrophy (DMD). In addition, one carrier couple was heterozygous for α-thalassemia, and the female partner was also a carrier of DMD.

## Discussion

In the present study, ECS is available to all couples who are planning to have children, either during pregnancy preparation or in the early stages of pregnancy, and we conducted the largest ECS to date in a city in Southwest China, Chongqing. We identified 5.34% of the couples and 0.37% of the female at risk for barrier offspring with a recessive disease in this area. In our data, the majority of patients were from the obstetrics department (*n* = 3378, 90.39%), including 948 couples and 1482 individuals. Of the 3378 subjects, 1298 women were screened without their partner. It was reported that the male partner uptake rate in the retrospective chart review was approximately 77% [13]. In our study, the male partner's acceptance rate was lower than theirs. Otherwise, we also included 359 individuals (101 couples) patients from the assisted reproduction department, of whom 168 individuals were positive for at least one condition (46.80%). As the individuals were mainly from Chongqing in the southwest of China, the results would be very important for relevant clinical work in the regions.

The carrier frequencies of top 10 conditions were consistent with the previous reports, the most common disorders screened was GJB2-autosomal recessive deafness 1A (*DFNB1A*) [14]. However, carrier frequency of *DFNB1* (12.95%) was higher than the previous published estimates (1.66%) [12]. In our ECS screening, p. Val37Ile (c.109 G > A) variant of *GJB2* gene is involved. Even if p. Val37Ile was removed, the frequency of DFNB1 is still high, about 2.62% (*n* = 98). Variants in p. Val37Ile are characterised by episodic incompleteness and late onset, i.e. some of the subjects with this pure or compound heterozygosity have no hearing abnormality or do not show hearing abnormality until a certain age. The previous studies have not counted this locus as a positive result, and in the present study, we analysed both the inclusion of the variant p.Val37Ile and the exclusion of this variant [12]. Furthermore, Zhang and colleague reported c.200T > G of IDS gene as the novel variant [15]. Hoshina et al. functional experiments were performed on the mutated site of this gene displayed enzyme activity that was higher than 5% of that of wild-type IDS [16]. Based on this, in our study, the c.200T > G variant in IDS gene was classified as likely pathogenic and included in the detected pool. Otherwise, as shown in Table 3, we detected a high carrier frequency of Wilson disease (2.89%), DFNB4 (2.09%), and SMA (2.06%). Wilson disease was investigated in a previous study, where a carrier frequency of 1.96% was reported [12], which is lower than our local data. Previously studies indicated that ethnicity, geographic region, level of economic development, disease spectrum and genetic background, testing methods and analysis pipeline, laboratory testing capacity, and the number of genetic diseases screened for were significantly associated with differences in test positivity rates between countries [17, 18]. In addition, consanguinity (marriage between close relatives) has been reported to be relatively common in some Asian populations, which may lead to an increased prevalence of autosomal recessive disease [19]. As the population we tested was mainly urban, with few minorities and a low prevalence of consanguineous marriages, it is more likely that the positive rates were due to the ECS screening strategies, we used to increase the positive rates.

In the present study, there are 5.34% (56/1048) of risk couples and 0.37% (9/2440) of female carriers in the data shown in Table 2. In the total data, we identified 42 heterozygous GJB2 mutation-positive couples. It has been reported that the penetrance of homozygotes in the c.109G > A variant was 17%, and the penetrance of compound heterozygotes was higher [20]. In couples with the GJB2 gene c.109G > A or c.235delC variant, prenatal diagnosis is not performed. Otherwise, we found that 10 subjects were homozygous for the c.109G > A mutation in the GJB2 gene. Seven of them did not complain of hearing loss or impairment. However, three of them complained of hearing impairment during daily work. Deafness gene mutations can occur in any population, regardless of ethnicity. However, some populations may have higher rates of certain mutations due to genetic factors or founder effects. For example, there are several genetic causes of hearing loss that is more common in certain populations, including those of Asian descent [21]. In addition, the frequency of carriers of recessive diseases varies widely among ethnic groups. For example, cystic fibrosis, caused by pathogenic variants in the CFTR gene, is the most common life-limiting AR disease affecting Caucasians, but is much lower in Asian Americans [22]. In contrast, β-thalassaemia, another common AR disease caused by deleterious variations in the HBB gene, is highly prevalent in Mediterranean, African, Central Asian, and Southeast Asian populations, but is much less prevalent among people of Caucasian descent [6]. These data suggest that in countries and regions with only one majority ethnic group, the recessive gene panels selected for carrier screening should be based on carrier frequencies in specific populations [23].

Notably, there are limitations to this study. First, it is a retrospective study in a single reporting centre in Chongqing, a large city in Southwest China. Second, most of the subjects were pregnant, even in the last weeks of pregnancy, which limited the time for the male partners to be examined and to decide on the outcome. Thirdly, because of the short implementation time of the ECS, we screened out a limited number of at-risk couples who had not yet been followed up. Therefore, genomic information generated by ECS should be handled securely and confidentially to protect patient privacy.

The ECS is a powerful strategy for preventing birth defects. Targeted NGS could screen 155 diseases at once with high efficiency. The selected diseases are customised for the Chinese population according to the guidelines, including known pathogenic and likely pathogenic variants. Indeed, a customised array cannot cover all diseases and genes. A negative result still cannot eliminate residual risks, so it is necessary to fully inform and clearly explain. Patients need to fully understand the implications of genetic testing before undergoing this ECS. Healthcare providers need appropriate education and training to effectively interpret and apply the results of ECS. Compared with microarray, NGS platform is more flexible, and it is convenient to adjust the detection range according to the situation in future. Moreover, our ECS panel detects 155 selected diseases. If microarray detects 155 diseases, its cost will be higher. The cost of customising microarray is higher than that of NGS panel, and service of customised NGS panel in local region is relatively easy to obtain. Lastly, NGS can detect many types of variation at one time, including SNP and CNV, etc. Furthermore, compared with the simplicity of WGS, our testing strategy targets genes with a higher incidence in the population to avoid over-testing and thus reduce the cost of the experiment, and also targets only the detection of known clearly pathogenic genes or possible pathogenic loci, which will facilitate efficient clinical genetic counselling and avoid increasing the stress and psychological burden on the patient as well as unnecessary costs of subsequent testing.

In conclusion, we report the first extended carrier screening of 3737 individuals in Chongqing. Among 1048 pairs, we found 56 pairs (5.34%) and 9 females (0.57%) at risk for autosomal or X-linked recessive disorders, with GJB2 autosomal recessive deafness 1A (DFNB1A) being the most common. Further prenatal diagnosis revealed a primary carnitine deficiency in one foetus of the carrier couple. Our clinical experience has shown that confidence in expanded carrier screening as a promising alternative and in genetic counselling is growing. In addition, accurate genetic diagnosis enables more informed decisions about family planning, prenatal care, and treatment options. Continually accumulating genomic databases facilitate the interpretation of novel genetic findings discovered through ECS.

### Supplementary Information

**Additional file 1.** Diseases and genes included in the carrier screening.

## Data Availability

The datasets used and analysed during the current study are available from the corresponding author on reasonable request.
